# Internationally trained pharmacists in Great Britain: what do registration data tell us about their recruitment?

**DOI:** 10.1186/1478-4491-7-51

**Published:** 2009-06-25

**Authors:** Ellen I Schafheutle, Karen Hassell

**Affiliations:** 1 Centre for Pharmacy Workforce Studies, Workforce Academy, School of Pharmacy and Pharmaceutical Sciences, The University of Manchester, Manchester, UK

## Abstract

**Background:**

Internationally trained health professionals are an important part of the domestic workforce, but little is known about pharmacists who come to work in Great Britain. Recent changes in the registration routes onto the Register of Pharmacists of the Royal Pharmaceutical Society of Great Britain may have affected entries from overseas: reciprocal arrangements for pharmacists from Australia and New Zealand ended in June 2006; 10 new states joined the European Union in 2004 and a further two in 2007, allowing straightforward registration.

**Aims:**

The aims of the paper are to extend our knowledge about the extent to which Great Britain is relying on the contribution of internationally trained pharmacists and to explore their routes of entry and demographic characteristics and compare them to those of pharmacists trained in Great Britain.

**Methods:**

The August 2007 Register of Pharmacists provided the main data for analysis. Register extracts between 2002 and 2005 were also explored, allowing longitudinal comparison, and work pattern data from the 2005 Pharmacist Workforce Census were included.

**Results:**

In 2007, internationally trained pharmacists represented 8.8% of the 43 262 registered pharmacists domiciled in Great Britain. The majority (40.6%) had joined the Register from Europe; 33.6% and 25.8% joined via adjudication and reciprocal arrangements. Until this entry route ended for pharmacists from Australia and New Zealand in 2006, annual numbers of reciprocal pharmacists increased. European pharmacists are younger (mean age 31.7) than reciprocal (40.0) or adjudication pharmacists (43.0), and the percentage of women among European-trained pharmacists is much higher (68%) when compared with British-trained pharmacists (56%). While only 7.1% of pharmacists registered in Great Britain have a London address, this proportion is much higher for European (13.9%), adjudication (19.5%) and reciprocal pharmacists (28.9%). The latter are more likely to work in hospitals than in community pharmacies, and all groups of internationally trained pharmacist are more likely to work full-time than British-trained ones. Adjudication pharmacists appear to stay on the Register longer than their reciprocal and European colleagues.

**Conclusion:**

Analysis of the Register of Pharmacists provides novel insights into the origins, composition and destinations of internationally trained pharmacists. They represent a notable proportion of the Register, indicating that British employers are relying on their contribution for the delivery of pharmacy services. With the increasing mobility of health care professionals across geographical borders, it will be important to undertake primary research to gain a better understanding of the expectations, plans and experiences of pharmacists entering from outside Great Britain.

## Background

There has been a growing shortage of health professionals worldwide, and this is no different in the United Kingdom. Here, the United Kingdom Border Agency issues a National Shortage Occupation List [1], which – among nurses and numerous medical disciplines – has included pharmacists (including preregistration pharmacists) and pharmacy technicians since 1998 [2]. Numerous factors have been identified as contributing to this shortage of pharmacists, and these relate to both supply and demand.

The Royal Pharmaceutical Society of Great Britain (RPSGB), which holds the Register of Pharmacists in Great Britain, defines Great Britain (GB) as England, Scotland and Wales, but not Northern Ireland (which is part of the United Kingdom) or the Isle of Man and the Channel Islands (part of the British Isles). The RPSGB accredits MPharm degree courses in Great Britain as well as Northern Ireland, but registration with either the RPSGB or the Pharmaceutical Society of Northern Ireland depends on where the preregistration year is undertaken and passed.

Throughout this paper, the authors refer to GB-trained pharmacists as those who have trained at a school of pharmacy in England, Scotland, Wales or Northern Ireland but have undertaken their 12-month preregistration training only in England, Scotland or Wales, so are registered with the RPSGB. The data analysis presented here is based on the RPSGB Register of Pharmacists and therefore applies only to England, Scotland and Wales: Great Britain.

The growing demand for pharmacists in Great Britain is related, in part, to an increase in their workload, with an increasingly elderly population, increased prescription volume, extended roles and new sectors of work, and the long opening hours of some types of community pharmacies all contributing to this [3,4]. The new pharmacy contract, which was implemented in 2005 and introduced payment for services as well as the more traditional reimbursement based on dispensing volume alone, is a further factor contributing to increased workload.

Conversely, a number of other factors affect the supply of pharmacists negatively, such as the increasing feminization of the workforce, an ageing workforce and the increase of part-time working among both men and women [5-7]. Another contributing factor is the emigration of pharmacists to other countries, since between about 10% and 11% of pharmacists registered in Great Britain are domiciled abroad (i.e. not in Great Britain) [2].

The shortage of pharmacists in Great Britain is being addressed in a number of ways. Changing the skill mix – in which other pharmacy team members, such as medicines counter assistants and pharmacy technicians, extend their roles and help increase efficiency – has been put to good effect in some pharmacy sectors [8,9]. The other approach has been to increase the number of pharmacy students; over the last 10 years, the capacity of pharmacists' training in Great Britain has been expanded, both by increasing the annual intakes of existing schools of pharmacy and by accrediting new schools of pharmacies [10,11].

Another way of increasing the number of registered pharmacists in Great Britain is through increasing the number of pharmacists who enter the Royal Pharmaceutical Society (RPSGB) Register of Pharmacists after an international qualification. Indeed, the 2005 report on future pharmacy workforce requirements published by the RPSGB recommends an increase of internationally trained pharmacists by 1% per year [12]. Other health care professions, such as doctors and nurses, have also seen similar increases in the number of internationally trained health professionals from overseas [13-18]. Active recruitment has also taken place for doctors and nurses, a strategy that is made easier if a health profession is listed as a shortage profession [1].

### Qualifying routes of entry to the Register of Pharmacists

Besides obtaining a pharmacy degree in a school of pharmacy in England, Scotland, Wales or Northern Ireland (the United Kingdom) and passing the requirements of a 12-month preregistration training course in England, Scotland or Wales (Great Britain), there are a number of other possible routes of registering with the RPSGB [19]. These apply to pharmacists who (1) have completed a pharmacy course that is comparable to those offered in the United Kingdom and (2) are registered, or eligible to register, in their country of training. To those who are both nationals of a European Economic Area (EEA) country (i.e. a European Union Member State, or Iceland, Liechtenstein, Norway and Switzerland) and have obtained such a qualification from an EEA country, the "system of automatic recognition of qualifications for specific professions" applies [20]. This route of entry to the GB Register is therefore relatively straightforward and does not require any further training or assessment. It is hereafter referred to as the European route of entry.

Pharmacists who are not EEA nationals or have obtained their qualification in a country outside the EEA must apply to the Adjudication Committee at the RPSGB, which will make a decision on the equivalence of their pharmacy qualification. If passed, these pharmacists can enrol on a 12-month Overseas Pharmacists Assessment Programme (OSPAP), which is presently offered at four universities in Great Britain. Once they have satisfied the OSPAP assessment, they need to complete a 12-month preregistration period with assessment, following which they can register with the RPSGB. Due to the requirement to apply to the Adjudication Committee at the beginning of this process, this entry route is commonly referred to as the "adjudication route".

Until recently, a third entry route was available to pharmacists from Australia and New Zealand, which was based on reciprocal arrangements between these countries and Great Britain. This required the production of relevant paperwork, such as the pharmacy degree certificate and information about registration in their country, and the completion of four weeks of supervised practice [21]. This route ceased on 30 June 2006, after a decision by the RPSGB Council that all applications from outside the EEA would be considered via the Adjudicating Committee process, thus increasing transparency and fairness [22]. This route now exists only for pharmacists who studied at a United Kingdom school of pharmacy, but undertook their 12-month preregistration training in Northern Ireland and, upon its successful completion, registered with the Pharmaceutical Society of Northern Ireland.

Even though there is some anecdotal evidence about active recruitment of pharmacists outside Great Britain, there is no published research evidence that has examined this. What is documented is that about 8% of pharmacists who live in Great Britain entered the Register after an international qualification [2]. It is not known if, and how, the removal of the reciprocal route of entry has affected the pharmacy workforce. Furthermore, besides Polish pharmacists making up the largest number of new European registrations in 2006 and 2007, overtaking new registrations from Spain [11,23], little else is known about the effect of the 12 new Member States of the European Union (EU) that joined in 2004 and 2007. Finally, it is not clear whether there are any differences between the pharmacists who enter the Register via different entry routes, how they compare to those who originally qualified in Great Britain, and whether any differences are likely to have an impact on workforce supply.

The aim of the analysis described here is to explore the GB Register of Pharmacists for differences in pharmacists' characteristics, depending on route of entry, i.e. after pharmacy training in Great Britain, or one of the three routes of entry available to internationally trained pharmacists, i.e. adjudication, reciprocal or European route of entry, and to describe other known characteristics of the internationally trained pharmacist workforce.

## Methods

The Register of Pharmacists lists all members of the RPSGB, and being a member is a prerequisite for being able to practise as a pharmacist in Great Britain. In the migration literature on doctors and nurses, "overseas" commonly refers to those health care professionals trained outside Europe. However, in this paper the term "internationally trained pharmacist" is used and refers to all pharmacists who did not obtain their original pharmacy qualification or registration in Great Britain.

The Register of Pharmacists contains information on individual pharmacists' date of birth, gender, ethnic background, location of registered address, date of first joining the Register, and practising status. It further records "overseas" status, i.e. whether pharmacists originally qualified in Great Britain or entered via the reciprocal, adjudication or European route. The RPSGB extracts a copy of this Register annually at the beginning of August, thus enabling longitudinal analysis of developments on the Register. The research team has obtained copies of these extracts since 2002. The anonymized August 2007 Register extract forms the basis for the analysis presented in this paper, but extracts from earlier years are sometimes used for longitudinal comparison.

Information from the 2005 Workforce Census of all pharmacists with an address in Great Britain is also included, as this provides the most recent and reliable data source on pharmacists' sector of work (i.e. community, hospital, or primary care = working for a primary care organization), and hours of work [24].

The Register extracts for 2006 and 2007 and data from the 2005 Census were merged, checked, cleaned and analysed using SPSS 14.0 (Statistical Package for the Social Sciences). Simple frequencies were produced, and to explore relationships between variables, Chi-square tests were performed. A one-way ANOVA was run to explore differences between mean ages.

Differences between pharmacists who trained in Great Britain and those who entered via the three routes detailed above (adjudication, reciprocal, European) were explored in relation to age, gender, ethnic origin, registered address (location) and workforce participation. Permanency of movement was also explored by looking at all (including non-GB) registered addresses of internationally trained pharmacists. Changes between the Register extracts of 2006 and 2007 were also analysed by identifying those pharmacists who joined between August 2006 and August 2007 ("joiners"), and those who left in the same period ("leavers").

## Results

### Registrations of internationally trained pharmacists in 2007, and developments in the past

In August 2007, a total of 47 962 pharmacists were registered with the RPSGB, and 43 262 (90.2%) had a registered address in Great Britain. Of those pharmacists with an address in Great Britain, 3802 (8.8%) were on the Register after an international qualification. Of those, the majority (40.6%) were from Europe, followed by those who had entered via the adjudication route (33.6%) and those who had entered through a reciprocal agreement (25.8%). Unless stated otherwise, all further analysis presented here will be based on registered pharmacists with an address in Great Britain, as they are the ones most likely to be available to the pharmacy labour market in Great Britain.

**Figure 1:**
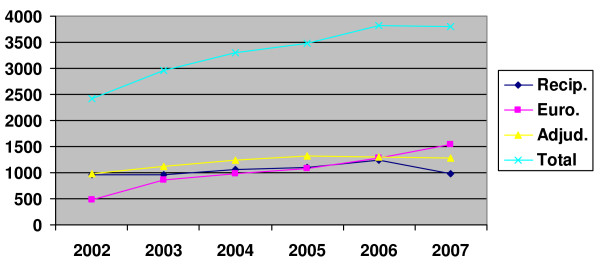
**Number of foreign trained pharmacists with a GB address, 2002–2007, overall and by route of entry**.

**Figure 2:**
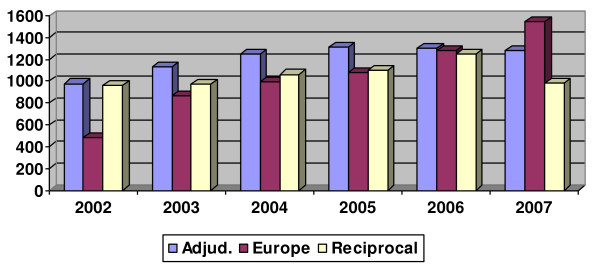
**Entry route for new pharmacist registrants between 2002 and 2007**.

When looking back over the five preceding years, 2007 was the first year since 2002 when the total number of internationally trained pharmacists with an address in Great Britain decreased (n = 3802 versus 3825 in 2006; 3482 in 2005; and 3292 in 2004 – see Figure 1). The percentage of those registered following adjudication has remained relatively constant over this period (between 2.7% and 3.3%), while the proportion of pharmacists entering via the European route has increased both in real and percentage terms. After annual increases in the number of those on the Register via reciprocal arrangements, particularly in 2006 before this entry route ended, their number decreased in 2007 (from 1245 [2.9%] in 2006 to 980 [2.3%] in 2007). These trends find further support when looking only at new registrations in the years from 2002 to 2007 (see Figure 2).

This trend is also confirmed when looking at the year of first registration of pharmacists with an address in Great Britain on the 2007 Register. The first pharmacist who joined the Register through the adjudication route did so in 1947, followed by the first pharmacist joining through the reciprocal route in 1949. As the legal foundation for the recognition of European pharmacy qualifications was laid only in 1985 [20], the first European pharmacist joined the Register in 1988. Nevertheless, the largest number of registrations through all three "overseas" entry routes has occurred in the last 10 years. This is particularly noticeable with European registrations, which were relatively slow in the first 15 years (until 2001), when a total of 217 pharmacists joined. The largest increases have occurred in the last three years, with 311 new entries in 2005 and 392 in 2006 alone, more than doubling European entries in the preceding three years.

### Demographics of internationally trained pharmacists with an address in Great Britain

The age distribution of pharmacists who entered after adjudication is relatively similar to that of GB-registered ones, if accounting for the fact that there are very few (2.1%) under the age of 30. The respective mean ages are 43.13 for Great Britain and 43.01 for adjudication pharmacists. Differences between means were compared using a one-way ANOVA, and least significant difference/Bonferroni confirms that significant differences exist between all group means except for GB-registered versus adjudication (F = 401.062; p ≤ 0.001). The mean age of pharmacists who entered via reciprocal arrangements is 40.04, but the age distribution shows marked differences, with large percentages being between 20 and 29 (34.1%) and between 30 and 39 years of age (29.5%). As European pharmacists started registering only in 1988, pharmacists who have entered via this route are younger than any other group (mean: 31.73); 44.5% are under 30, and 44.0% under 40 years of age.

When specifically looking at changes between the 2006 and 2007 Register extracts, it can be seen that the majority of GB-trained leavers (total n = 820) fell into the 60 to 69 (32.9%) and 70 and over age brackets (40.4%). Only 12 adjudication pharmacists left, and seven of them were over 69. About a fifth of the 89 leavers who had originally joined through a reciprocal arrangement left aged 60 to 69 (11.2%) or 70 and over (10.1%), but the majority were young, between 22 and 29 (49.4%) and 30 and 39 (23.6%). As there are very few older European pharmacists on the Register, it is unsurprising that of the 65 who left, 49.4% were aged 22 to 29, and 46.2% 30 to 39.

Among those with a GB address, women outnumber men slightly on the Register (56.3%). Those who entered the Register via adjudication have a similar percentage of women (55.3%) to those who are GB-trained (55.8%). Reciprocal pharmacists have a slightly higher percentage of women (56.3%), whereas as many as 67.8% of European pharmacists are women. These higher percentages appear to be due mainly to the larger number of younger pharmacists, where both European and GB-registered pharmacists show similar percentages (66.0% and 69.9% female among 22-to-29-year-olds, respectively, and 63.1% and 68.5% among those aged 30 to 39).

The ethnic origin of pharmacists is also recorded on the Register. This information is missing for 12.1% of those with a GB address, but analysis still provides useful insights. Some 72.7% of GB-registered pharmacists are white and 20.6% are Asian. The percentage of white pharmacists among those joining via reciprocal arrangements is somewhat higher (78.7%), whereas by far the largest proportion of European pharmacists is white (94.2%). The largest group among pharmacists who have joined the Register through adjudication are black (39.2%). Table 1 shows more detail.

**Table 1:** Ethnic origin of pharmacists on 2007 Register with a GB address

	**GB-reg %**	**Adjudication %**	**Europe %**	**Reciprocal %**	**Total**
White	72.7	22.0	94.2	78.7	72.2

Asian	20.6	27.7	2.5	5.4	19.9

Black	2.6	39.2	1.5	1.2	3.5

Chinese	2.3	1.1	0.2	10.2	2.4

Mixed	0.7	1.7	0.9	1.1	0.8

Other	1.0	8.3	0.8	3.5	1.2

Total n (excluding those missing)	34 873	1017	1283	834	38 007

As pharmacists' ethnic origin is likely to be related to the ethnic make-up of the country of training, it would be useful to have this information available. Unfortunately, the country of training is not recorded on the Register. This information is recorded elsewhere, though, as the Society reports these data for new entries to the Register in its own annual Register reports [11,23].

Since 2005, the GB Register of Pharmacists classifies pharmacists into practising and non-practising. Practising pharmacists are defined as those who "undertake any work in, or gives advice in relation to, the science of medicines or the practice of pharmacy or healthcare" [25]. This therefore comprises all pharmacists working in the traditional settings of community, hospital and, more recently, primary care pharmacy, but the definition is wider and also applies to those who teach pharmacy, for example. It thus applies to the majority (89.2%) of registered pharmacists with a GB address. However, internationally trained pharmacists are significantly more likely to be registered as practising than are GB-trained pharmacists (88.7%). Those who entered after adjudication have the highest percentage of practising status (96.7%), followed by European (95.3%) and reciprocal pharmacists (89.8%). All analysis presented in this paper includes all practising and non-practising pharmacists, as the latter are in the minority and can re-enter the practising Register.

### Geographical distribution within Great Britain

Analysis of pharmacists' registered addresses in Great Britain shows that internationally trained pharmacists are more likely to live or work in England than in Scotland or Wales. While 83.3% of GB-trained pharmacists have a registered address in England, 94.5% of internationally trained ones do. Furthermore, independent of route of entry onto the Register, internationally trained pharmacists are more likely to live in London than are GB-trained pharmacists (7.6%), and this is most pronounced for those who had entered via a reciprocal arrangement (28.9%), followed by adjudication (19.5%) and European (13.9%) pharmacists.

### Return migration to country of training

For workforce planning purposes it is important to understand how long internationally trained pharmacists are likely to remain in Great Britain. Despite otherwise focusing analysis on pharmacists with a GB address, this section does include an exploration of internationally trained pharmacists with an address outside Great Britain. Of the internationally trained pharmacists on the 2007 Register, just over half (50.5%) of those who joined through reciprocal agreements have registered addresses outside Great Britain. Over a fifth (21.9%) of those originally registered in Europe live outside Great Britain. Of those pharmacists who came onto the Register via an adjudication route, only 9.9% have an address outside Great Britain, a figure that is not much higher than that of GB-trained pharmacists (7.2%).

Table 2 shows the changes of pharmacists' registered addresses between August 2006 and August 2007, split by the route of entry (including GB-trained). In just one year, only a relatively small percentage (0.7%) of GB-trained pharmacists living in Great Britain in 2006 moved to an address outside Great Britain in 2007. The highest percentage of movement to a non-GB address was observed for pharmacists who had entered via the reciprocal arrangement (19.1%), followed by European-entry pharmacists (8.2%). These findings suggest that a large percentage of those entering via reciprocal (in particular) and European arrangements stay in Great Britain for only a limited period of time, which lends further support to the analysis presented in the preceding paragraph.

**Table 2:** Movement of address (GB ←→ overseas) between 2006 and 2007

**Route of entry**	**Address in 2007**	**GB address in 2006**	**Overseas address in 2006**
GB-trained	GB	37 639 (99.3%)	122 (4.3%)
	
	Overseas	253 (0.7%)	2726 (95.7%)

Adjudication	GB	1270 (98.3%)	4 (3.3%)
	
	Overseas	22 (1.7%)	117 (96.7%)

Europe	GB	1115 (91.8%)	77 (24.1%)
	
	Overseas	99 (8.2%)	243 (75.9%)

Reciprocal	GB	936 (80.9%)	31 (3.7%)
	
	Overseas	221 (19.1%)	802 (96.3%)

The above analysis suggests that those who register after an international qualification may be returning to their country of training. As the Register does not hold information on the country of training (as previously mentioned), this is explored by looking at the GB Register entry arrangements that the country of non-GB residence would fall under. The majority of pharmacists with a registered address outside Great Britain (between 76.3% and 95.2%, depending on route of entry) now live in a country with the same qualification requirements for entry onto the GB Register as their own route of entry. The majority of pharmacists who had entered via adjudication and now have an address in a country requiring adjudication for entry to the GB Register live in the United States (40.6%), South Africa (24.5%) or Canada (7.5%). Of those who had joined via reciprocal arrangements and now live in a country with these arrangements (n = 808), 72.3% live in Australia and 27.7% in New Zealand. Those who had entered the Register from Europe and now have a European address (n = 415) live in Spain (33.7%), Germany (12.3%), Italy (9.6%) or Poland (8.2%).

### Link with census information on working patterns

The last census of all pharmacists with a GB address dates back to 2005 [24], so it was a couple of years out of date at the time of the 2007 Register extract. Nevertheless, in the absence of more recent data it still provides some useful information on pharmacists' work patterns, and whether they differ depending on route of entry for those with a registered address in Great Britain.

In the 2005 census, pharmacists were asked about the sector of their main job (11.6% said they had two or more jobs in different sectors). Those who had entered through adjudication were significantly more likely to work in community pharmacy (76.3%) than those who were GB-trained (64.9%), whereas they were less likely to work in hospital pharmacy (16.9% versus 21.1%). This finding was the reverse for pharmacists who had joined the Register through reciprocal arrangements; they were significantly less likely to work in community pharmacy (57.2%) and more likely to work in hospital pharmacy (30.0%). Even though the percentage of European pharmacists working in community pharmacy (62.8%) was similar to that of GB-trained pharmacists, a significantly higher percentage worked in a hospital (29.3%). This may, at least in part, be explained with relatively less employment of European pharmacists in primary care (1.6%; versus adjudication: 2.4%; reciprocal: 4.6%; GB-trained: 6.1%).

The 2005 Census also asked about total hours worked by respondents. All internationally trained pharmacists were more likely to work full-time (≥ 33 hours) (81.3% for adjudication up to 89.3% of European pharmacists) than GB-trained ones (67.7%). Pharmacists who had entered via the adjudication route stated they worked the longest hours, with 13.3% working more than 48 hours, and 27.8% working between 41 and 48 hours.

Finally, one further item that is recorded in the 2007 Register is whether a pharmacist is a supplementary or independent prescriber. The numbers among all pharmacists are still low, with only 1191 (3.0%) GB-trained pharmacists recorded as a supplementary prescriber, and only 140 (0.4%) recorded as an independent prescriber. Nevertheless, there are significantly (p < .001) fewer supplementary prescribers among internationally trained pharmacists. The highest percentage of supplementary prescribers among internationally trained pharmacists is found among those who entered via the adjudication route (n = 26; 2.0%), followed by 14 (1.4%) reciprocal and 10 (0.6%) European pharmacists. There are only two independent prescribers who had entered via the adjudication route.

## Discussion

This paper provides novel insights into the country of training, composition and destination of internationally trained pharmacists on the GB Register of Pharmacists. Three routes of entry for internationally trained pharmacists were available prior to 2006. Two different routes of entry are now available, which depend on the country of training, and they differ significantly in the ease and cost incurred by the pharmacist when trying for GB registration. By drawing comparisons between pharmacists who have joined the Register through the different routes, this paper identifies a number of important differences between pharmacists who have joined via adjudication, reciprocal or European arrangements. These have important implications for the GB pharmacy workforce, the relevance of which for short-term and long-term planning is discussed here. Given that the relatively straightforward reciprocal route of entry for pharmacists from Australia and New Zealand ended in June 2006, around the time of enlargement of the European Union, such comparisons and consideration of migration patterns are highly relevant and topical.

Pharmacists who originally trained outside Great Britain constitute a reasonable proportion of the GB Register (8.8% in 2007). Due to a shortage of pharmacists in Great Britain, a recommendation was made to increase the number of internationally trained pharmacists [12]. However, the analysis presented here shows that the number of internationally trained pharmacists living and working in Great Britain decreased for the first time in 2007. In the main this appears to be due to the reduction of pharmacists who were on the Register after reciprocal arrangements, as this route of entry ceased in June 2006. Nevertheless, the numbers of pharmacists who have entered after adjudication have continued to increase at rates similar to previous years, whereas registrations of European pharmacists have increased considerably since the joining of 12 new European Union Member States in 2004 and 2007.

Adjudication pharmacists appear to retain a registered address in Great Britain the longest and, apart from their ethnic origin (mostly black rather than white or Asian), appear most similar to those pharmacists who originally qualified in Great Britain. Not only do they have similar age and gender profiles, they are also more likely to work full-time than their GB-trained counterparts, and they appear to have similarly low percentages moving to an address outside Great Britain. The pharmacists who enter via the adjudication route perhaps stay on the Register longer and remain part of the pharmacy workforce for longer, possibly because their investment in time and money is more substantial than for pharmacists entering through other routes. Furthermore, work permits would be required for many non-EEA pharmacists when they first arrive in Great Britain, and this may further constrain their mobility. This however, would need to be substantiated through new primary research.

Reciprocal and European-entry pharmacists tend to be young, but also leave the Register relatively young, suggesting that many of them stay for only a few years, possibly before returning to their country of training. Nevertheless, their contribution to the GB pharmacy workforce is sizeable year on year, representing, as they do, 2.3% and 3.6% of the Register, respectively.

The impact of the abolition of the reciprocal route of entry for pharmacists from Australia and New Zealand may not become clear for a number of years, but this study shows that the number of reciprocal pharmacists has already decreased considerably (by 21%) in just 12 months. Further analysis suggests that, during their time in Great Britain, these pharmacists are most likely to have addresses in England, and London in particular, and they are most likely to work in hospital pharmacy. The impact would therefore be expected to be most noticeable for recruitment into junior hospital pharmacy positions in London. This may add further to the already high rate of unfilled vacancies, which is particularly high (17%) among junior hospital pharmacists. [26] This would merit further exploration.

With the considerable increase in European-entry pharmacists, they may be replacing pharmacists from Australia and New Zealand. According to analysis presented here, they are also (albeit not as much as reciprocal pharmacists) more likely to be working in London and in hospital pharmacy than either GB-trained or adjudication pharmacists.

What this study cannot provide is analysis that goes beyond the exploration of patterns of pharmacists' characteristics and movement. No data are available that provide further insight into the reasons why internationally trained pharmacists decide to come to Great Britain, and how they differ depending on their route of entry. What is also not known is whether their expectations are met, how they manage while they are in Great Britain, and whether their intention to return to their country of training (or elsewhere) is influenced by experiences while they reside in Great Britain.

Nor is there research into the ethical obligations and responsibilities of pharmacy employers, in particular their role in inducing the exit of qualified pharmacists from source countries into Great Britain. The impact on the health care systems in those countries when qualified pharmacists migrate has also not been investigated. There is little published research that has specifically looked at internationally trained pharmacists, but considerably more is known about other health professionals, particularly doctors and nurses who work in the National Health Service (NHS) after an international qualification.

It is known that GB-trained pharmacists who migrated overseas have done so for lifestyle and economic reasons, to better their career prospects, accompany their partner or spouse, or to return to their home country following GB training [2]. These reasons are echoed in studies with other health professionals, particularly doctors and nurses [27-30]. Importantly, reasons for migration appear to differ depending on whether migration occurs from a developed or developing country, and these differences may be similar among internationally trained pharmacists in Great Britain and would therefore need to be explored separately.

Some studies have also reported on overseas doctors' and nurses' experiences of life and work in the United Kingdom as a whole. Problems that are faced by overseas health professionals are those of cultural integration and/or difference; language has also been identified as a potential issue [31,32].

Studies with overseas nurses (and doctors) have further identified problems of exploitation, discrimination and racism [33]. These manifest themselves on a day-to-day basis, where nurses feel exploited because managers use them to cover undesirable shifts [34]. They also manifest themselves in the longer term, where nurses experience unequal opportunities for skill development and training as well as slower career progression than their United Kingdom-trained counterparts [35-37].

In contrast, a questionnaire study with international health and social care workers in the United Kingdom found that respondents identified greater access to continuing professional development (CPD), more career opportunities and a well-defined career structure as what was attractive about working in the United Kingdom [38]. Further studies, most likely of a qualitative nature, would need to be conducted to allow in-depth exploration of some of these issues within different groups of internationally trained pharmacists. For example, whether the finding from this study that pharmacists joining via the adjudication route were less likely to work in hospital pharmacy, or be qualified as supplementary or independent prescribers, is due to choice or some form of disadvantage or discrimination, may be worth follow-up research.

## Conclusion

This study has provided a greater appreciation of the size and composition of the internationally trained pharmacist workforce; it has also quantified the scale of pharmacist migration to Great Britain and identified recent trends and changes relating to the countries of training of the pharmacists. While internationally trained pharmacists constitute a not-insignificant proportion of the GB Register of Pharmacists, and while their contribution is likely to be helping employers meet service demands, it is also clear from this analysis that Great Britain does not rely on internationally trained pharmacists to the same extent as it does on internationally trained doctors or nurses. In fact, pharmacy in Great Britain may be relying more on European sources than other health care professions.

Furthermore, this study has identified gaps in knowledge about internationally trained pharmacists, not least our lack of understanding about the impact of movement on Great Britain as a receiving country and on the countries that are losing trained pharmacists. The study identified differing lengths of stay, which appear temporary for many European and reciprocal pharmacists, and varying geographical distributions depending on entry route, where many European and reciprocal pharmacists have registered GB addresses in London.

These observations have implications for the make-up of the GB pharmacist workforce and the role of internationally trained pharmacists within this. These data provide important insight for future planning of the pharmacist workforce, and related developments will require monitoring over time.

However, further primary collection of both qualitative and quantitative data will be required to explore the reasons for the patterns and observations presented. This will have to include pharmacists who have entered Great Britain following all three routes of entry, and ought to include respondents of different gender, ages and lengths of stay.

## Competing interests

The authors declare that they have no competing interests.

## Authors' contributions

ES and KH conceived of the study, and participated in its design, analysis and helped draft the manuscript. ES performed the statistical analysis. Both authors read and approved the final manuscript.
